# Dietary *Broussonetia papyrifera* flavonoids affect broiler muscle fiber development, meat quality, antioxidant capacity, and muscle metabolomic profiles

**DOI:** 10.3389/fvets.2026.1891551

**Published:** 2026-08-06

**Authors:** Min Yang, Enze Zhang, Bingyu Zhu, Hailong Wang, Xing Zhou, Mengling Peng, Jie Zhou, Jianbo Cheng, Xiuheng Xue, Juhua Wang

**Affiliations:** 1 College of Veterinary Medicine, Anhui Agricultural University, Hefei, Anhui, China; 2 College of Animal Science and Technology, Anhui Agricultural University, Hefei, Anhui, China; 3 College of Food and Nutrition, Anhui Agricultural University, Hefei, Anhui, China

**Keywords:** antioxidant capacity, broiler, *Broussonetia papyrifera* flavonoids, meat quality, metabolomics, muscle fiber

## Abstract

**Background:**

Intensive and antibiotic-free broiler production requires nutritional strategies that support meat quality and redox stability. *Broussonetia papyrifera* flavonoids (BPF) have antioxidant and metabolic regulatory activities, but their effects on broiler muscle development and muscle metabolomic profiles remain unclear.

**Methods:**

A total of 240 healthy 1-day-old Arbor Acres broilers were randomly assigned to four dietary treatments for 42 days: a basal diet without BPF (control) or basal diets supplemented with 100, 200, or 300 mg/kg BPF (groups I–III). Meat quality, muscle morphology, antioxidant indices, and non-targeted LC-MS/MS metabolomic profiles were evaluated in pectoral and thigh muscles.

**Results:**

BPF improved selected water-holding capacity indices by reducing pectoral cooking loss and thigh drip loss (*P* < 0.05), with no significant effect on shear force (*P* > 0.05). BPF also altered pH and color traits, reduced muscle fiber diameter, increased fiber density, enhanced T-AOC, T-SOD, and GSH-Px activities, and decreased MDA content, with the most consistent responses observed at 200 mg/kg. Non-targeted metabolomics of the control and 200 mg/kg groups identified 75 differential metabolites in pectoral muscle and 65 in thigh muscle (*P* < 0.05). Enriched pathways were mainly associated with amino acid metabolism, phenylpropanoid biosynthesis, lipid metabolism, cholesterol metabolism, fatty acid digestion and absorption, and oxidative stress-related metabolic regulation.

**Conclusion:**

Dietary BPF, particularly at 200 mg/kg, improved selected broiler meat quality, muscle fiber traits, and antioxidant status. The metabolomic findings provide exploratory evidence of muscle-specific metabolic remodeling that requires targeted validation.

## 1 Introduction

The poultry industry has expanded rapidly under intensive production systems, while high-density rearing, environmental stress, and restrictions on antibiotic use continue to challenge broiler health and meat quality. Phytogenic feed additives have therefore been investigated as green, residue-free nutritional strategies for supporting animal performance and product quality (1). *Broussonetia papyrifera* (paper mulberry), which is widely distributed in Asia, contains proteins, amino acids, minerals, flavonoids, terpenoids, phenolic compounds, and other bioactive constituents (2). Among these constituents, flavonoids are of particular interest because of their antioxidant, anti-inflammatory, antimicrobial, and metabolic regulatory activities (3, 4).

Dietary flavonoids can improve physiological resilience and meat-related traits in poultry. High-flavonoid corn improved broiler performance and resistance to enteric challenge (5), quercetin supplementation improved meat stability and immunological responses (6), maternal mulberry-leaf flavonoids promoted skeletal muscle development in offspring chickens (7), and baicalein enhanced muscle proliferation-related responses under heat stress (8). These findings suggest that flavonoid-rich feed resources may regulate broiler muscle growth and meat quality. However, the postnatal effects of *Broussonetia papyrifera* flavonoids (BPF) on broiler muscle fiber development and muscle metabolism remain insufficiently defined.

Oxidative stress contributes to lipid peroxidation, impaired cellular function, and reduced meat quality (9, 10). Skeletal muscle determines the edible yield and consumer acceptance of broilers, and muscle fiber diameter, fiber density, water-holding capacity, pH, shear force, and color are key indicators linking muscle development to meat quality (11). Because flavonoids can act as exogenous antioxidants and may regulate endogenous antioxidant enzymes, BPF may improve meat quality through coordinated effects on oxidative balance, muscle fiber development, and muscle metabolism. The present study therefore evaluated the effects of dietary BPF supplementation at 100, 200, and 300 mg/kg on meat quality, muscle morphology, antioxidant capacity, and non-targeted metabolomic profiles in broiler pectoral and thigh muscles, with the aim of identifying an effective dietary level and exploring potential metabolic associations underlying BPF-related changes in muscle quality.

## 2 Materials and methods

### 2.1 Ethical permission

All animal procedures were reviewed and approved by the Animal Ethics Committee of Anhui Agricultural University (approval No. KJLLXM2023067). The experiment was conducted in accordance with institutional guidelines for the care and use of experimental animals and reported in accordance with the ARRIVE guidelines.

### 2.2 Materials

BPF were supplied by Xi‘an Rui Ying Biotechnology Co., Ltd. (Xi'an, Shaanxi, China; Batch No.: 20200223882). According to the supplier-provided quality-control information, the product contained 40.26 ± 0.23% total flavonoids. The identified constituents were quercetin (34.15%), kaempferol (21.95%), isorhamnetin (15.07%), stachyose (12.20%), naringenin (7.32% and 4.88%), luteolin (2.44%), and other components (1.99%). The same product batch was used throughout the feeding trial, and the powder was stored in a dry, light-protected environment before diet preparation.

### 2.3 Experimental broilers, design, and management

A total of 240 healthy 1-day-old Arbor Acres broilers from Jiangsu Jinghai Poultry Group (Jiangsu, China) were randomly allocated to four dietary treatments in a completely randomized design: control, groups I–III. Each treatment included five replicates with 12 broilers per replicate. The control group received the basal diet without BPF, whereas groups I–III received basal diets supplemented with 100, 200, and 300 mg/kg BPF, respectively. The trial lasted 42 days. Broilers were kept under continuous light and standard broiler management, with ad libitum access to feed and water. BPF powder was premixed and blended into the basal diet to ensure uniform intake. The basal diet was formulated according to NRC (1994) recommendations (12), and its ingredient composition and nutrient levels are shown in Table 1.

**Table 1:** Composition and nutrient levels of the basal diet (air-dry basis, %).

Item	Content	
	0–21 d	22–40 d
Material		
Corn	62.5	65.5
Soybean meal	21.0	18.0
Bran	3.0	3.0
Fish meal	3.0	3.0
protein powder	5.0	5.0
Yeast powder	2.0	2.0
CaHPO_4_	1.2	1.2
Limestone powder	1.0	1.0
NaCl	0.3	0.3
^1^Premixture	1.0	1.0
Total	100	100
Nutritional level		
Crude protein	23.0	20.0
Coarse fiber	7.0	9.0
Coarse ash	9.0	10.0
Calcium	1.40	1.20
Total phosphorus	0.70	0.60
Methionine	0.50	0.45

The premixture provided the following per kg of diets: VA 8000 IU, VD3 1228 IU, VE 15 IU, VK3 3.0 mg, VB1 1.3 mg, VB2 3.1 mg, VB6 1.2 mg, calcium pantothenate 13.4 mg, choline chloride 500 mg, biotin 0.11 mg, niacin 25 mg, folic acid 0.68 mg, VB12 0.03 mg, Fe 120 mg, Cu 10 mg, Zn 130 mg, Mn 100 mg, I 0.3 mg, Se 0.3 mg.

### 2.4 Sample collection

On day 42, three broilers per replicate (15 birds per treatment) were randomly selected and euthanized by jugular exsanguination. Pectoral and thigh muscles were collected immediately. A portion of each fresh muscle sample was used for pH, drip loss, cooking loss, shear force, and meat color measurements. A tissue block of at least 3 × 2 × 2 cm was fixed in 4% paraformaldehyde for histological analysis. The remaining pectoral and thigh muscle samples were divided into subsamples and stored at −80 °C for antioxidant capacity and metabolomic analyses.

### 2.5 Meat quality measurement

For drip loss, right pectoral and thigh muscle samples from 15 broilers per group were weighed as W1, placed in sealed bags, stored at 4 °C for 24 h, blotted to remove surface moisture, and reweighed as W2. Drip loss was calculated as (W1 - W2)/W1 × 100%. For cooking loss, samples from 15 broilers per group were weighed as W3, cooked in an 85 °C water bath until the internal temperature reached 70 °C, cooled for 20 min, and reweighed as W4. Cooking loss was calculated as (W3 - W4)/W3 × 100%. Fifteen samples were measured per group, with triplicate measurements for each sample.

The pH of pectoral and thigh muscles were measured after 24 h storage at 4 °C using a portable digital pH meter (Testo 206, Testo Instruments International Trading Co., Ltd., Shanghai, China). The pH probe was calibrated before use and rinsed with distilled water between measurements. Fifteen samples were measured per group, with triplicate measurements for each sample.

Fresh pectoral and thigh muscle samples free of visible tendon, fat, and fascia were trimmed to approximately 1 cm width and 0.5 cm thickness. Shear force was measured using a C-LM2 muscle tenderness meter (Guangzhou Runhu Instrument Co., Ltd., Guangzhou, China) with the blade oriented perpendicular to the muscle fiber direction and expressed in Newtons (N). Fifteen samples were measured per group, with triplicate measurements for each sample.

Meat color was measured on the fresh cut surface using a CR-400 colorimeter (Hangzhou Color Spectrum Technology Co., Ltd., Hangzhou, China), and lightness (L^*^), redness (a^*^), and yellowness (b^*^) were recorded. Fifteen samples were measured per group, and each sample was measured in triplicate.

### 2.6 Muscle morphology analysis

Pectoral and thigh muscle samples fixed in 4% paraformaldehyde were dehydrated, paraffin-embedded, sectioned at 5 μm thickness, stained with hematoxylin and eosin, and mounted with Permount (Suyi Chemical Reagent Co, Ltd., Shanghai, China). Sections were observed using an Olympus microscope (Olympus, Japan). Muscle fiber diameter and density were quantified using Slide Viewer image analysis software (3DHISTECH, Inc.). Six broilers per group were analyzed. For each sample, three non-overlapping fields of 100,000 μm^2^ were measured, and mean values of muscle fiber diameter and density were calculated.

### 2.7 Determination of muscle antioxidant capacity

Pectoral and thigh muscle samples stored at −80 °C were weighed and homogenized on ice with phosphate-buffered saline at a tissue weight (g) to PBS volume (mL) ratio of 1:9 to prepare 10% tissue homogenates. Total antioxidant capacity (T-AOC; A015-1), total superoxide dismutase (T-SOD; A001-1-1), glutathione peroxidase (GSH-Px; A005), and malondialdehyde (MDA; A003-2-2) were determined using commercial assay kits from Nanjing Jiancheng Bioengineering Institute (Nanjing, China) according to the manufacturer's protocols. Six broilers per group were analyzed, and each sample was measured in triplicate.

### 2.8 Non-targeted metabolomic analysis

Based on the integrated meat quality, muscle morphology, and antioxidant results, group II (200 mg/kg BPF) was selected for non-targeted metabolomic comparison with the control group. For each muscle type, three biological replicates were randomly selected from the control and group II treatments.

Untargeted metabolomic profiling was performed using ultra-high-performance liquid chromatography coupled to hybrid quadrupole-Orbitrap tandem mass spectrometry (UHPLC-Q-Orbitrap MS/MS). The chromatographic separation was carried out on a Thermo Vanquish UHPLC system, and mass spectra were acquired with a Q Exactive Plus mass spectrometer (Thermo Fisher Scientific, USA). Chromatographic separation was achieved on a reversed-phase C18 column (2.1 × 100 mm, 1.7 μm) maintained at 40 °C. The mobile phases consisted of water containing 0.1% formic acid (mobile phase A) and acetonitrile containing 0.1% formic acid (mobile phase B), delivered at a flow rate of 0.3 mL/min under a 20-min gradient elution program. The injection volume was 5 μL. Mass spectrometric detection was performed using an electrospray ionization (ESI) source in positive ion mode, and data were acquired in full-scan MS mode with data-dependent MS/MS acquisition to obtain precursor and fragment ion information for metabolite annotation. Key source parameters, including capillary voltage, gas temperature, drying gas flow, nebulizer pressure, and collision energy, were optimized before sample analysis.

All raw chromatographic and mass spectral data were processed using MassHunter Profinder software (Version 12.0, Agilent Technologies, Inc., California, USA) for peak detection, deconvolution, retention-time alignment, and feature extraction. Pooled quality-control (QC) samples, prepared by mixing equal aliquots from all biological samples, were injected periodically throughout the analytical sequence to monitor system stability, signal reproducibility, and analytical drift. Solvent blanks prepared with 53% methanol-water were analyzed in parallel to remove background and carryover signals. Metabolite features were annotated based on accurate mass-to-charge ratio (m/z), retention time, isotope pattern, and MS/MS fragment information by matching against available spectral databases. Subsequent preprocessing, including feature filtering, missing-value imputation, normalization, and scaling, was performed using R and Python before multivariate statistical analysis, differential metabolite screening, and pathway enrichment analysis. The workflow followed standard untargeted LC-MS-based metabolomics procedures (13).

Identified metabolites were annotated using the KEGG, HMDB, and LIPID MAPS databases. Differential metabolites were screened using VIP > 1, *P* < 0.05, and fold change >= 2 or < = 0.5 as default criteria. The metabolomics workflow was non-targeted and non-quantitative; therefore, pathway enrichment was used to identify biological patterns requiring further targeted validation.

### 2.9 Statistical analysis

Data were analyzed by one-way analysis of variance using SPSS 21.0 software (SPSS Inc., Chicago, IL, United States). Results are presented as mean ± SEM. When the overall treatment effect was significant, treatment means were compared using Duncan's multiple range test. For meat quality, muscle morphology, antioxidant indices, and metabolomic profiling, the individual muscle sample was used as the experimental unit, as appropriate for the measured endpoint; each bird was sampled once for each tissue measurement. Differences were considered significant at *P* < 0.05 and highly significant at *P* < 0.01.

## 3 Results

### 3.1 Effects of BPF on water-holding capacity, pH, and shear force

**Figure 1:**
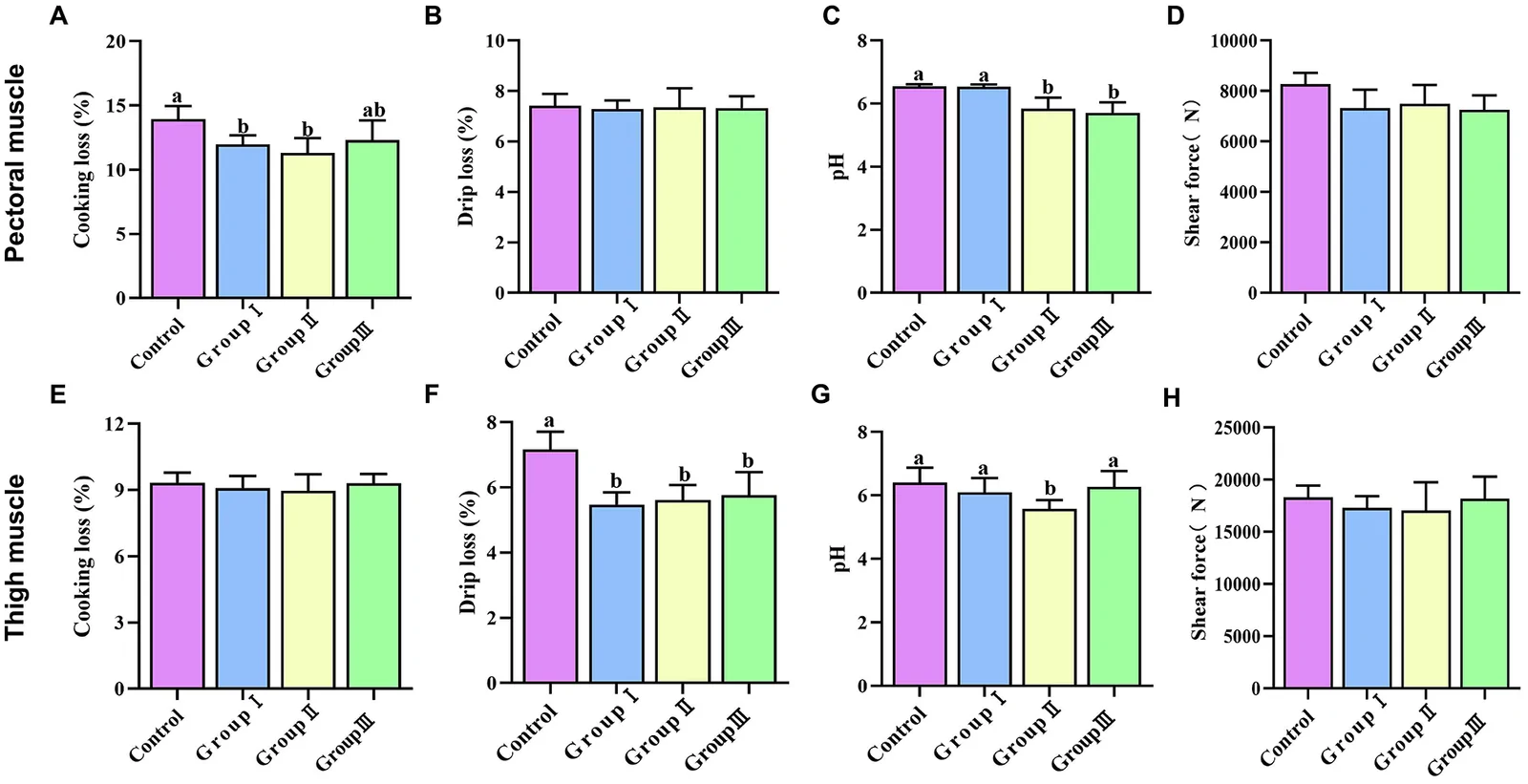
Effects of BPF on water-holding capacity, pH and shear force of broiler muscles. **(A–D)** Cooking loss, drip loss, pH and shear force of pectoral muscle. **(E–H)** Cooking loss, drip loss, pH and shear force of thigh muscle, respectively. Fifteen broilers were sampled for analysis of muscle quality in each group. Error bars represent SEM of 15 independent samples. Bars with the same letter or no letter show no significant difference (*P* > 0.05); different letters indicate significant differences (*P* < 0.05), *n* = 15.

Dietary BPF affected selected water-holding capacity indices in broiler muscles (Figure 1). In pectoral muscle, cooking loss was significantly lower in groups I and II than in the control group (*P* < 0.05), whereas III did not differ from the control group (Figure 1A). Drip loss in pectoral muscle and cooking loss in thigh muscle were not significantly affected by BPF supplementation (*P* > 0.05; Figures 1B–E). In thigh muscle, drip loss was significantly reduced in groups I, II and III compared with the control group (*P* < 0.05; Figure 1F).

BPF also altered muscle pH in a muscle- and dose-dependent manner. Pectoral muscle pH was significantly lower in groups II and III than in the control and group I (*P* < 0.05; Figure 1C). In thigh muscle, pH was significantly lower in group II than in other groups (*P* < 0.05), whereas groups I and III did not differ from the control (*P* > 0.05; Figure 1G). Shear force in pectoral and thigh muscles did not differ among treatments (*P* > 0.05; Figures 1D–H).

### 3.2 Effects of BPF on pectoral and thigh muscle color

**Figure 2:**
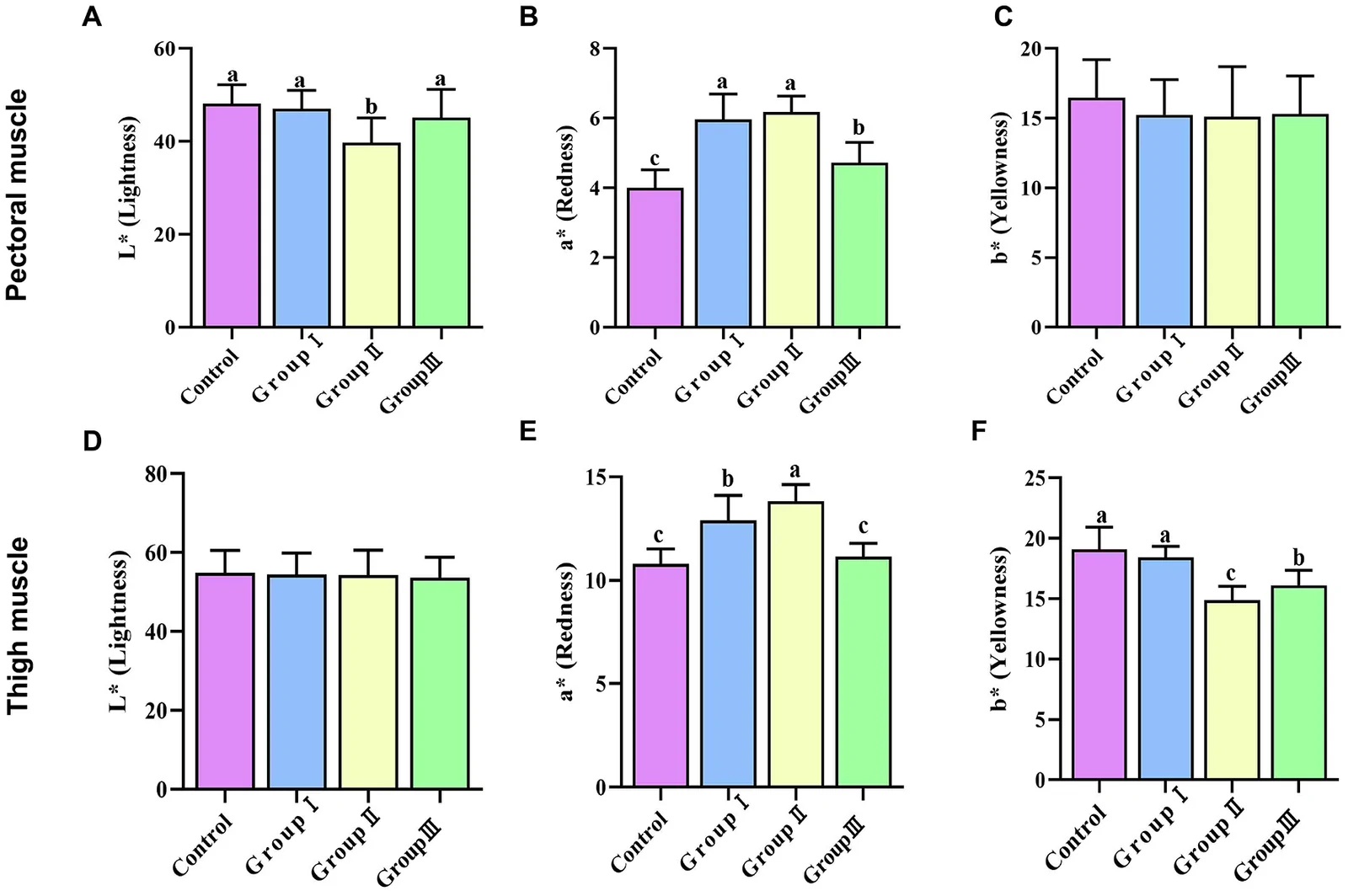
Effects of BPF on broiler muscle color. **(A–C)** L*, a*, and b* values of pectoral muscle, respectively. **(D–F)** L*, a* and b* values of thigh muscle, respectively. L*: lightness; a*: redness; b*: yellowness. Fifteen broilers were sampled for analysis of muscle quality in each group. Error bars represent SEM of 15 independent samples. Bars with the same letter or no letter show no significant difference (*P* > 0.05); different letters indicate significant differences (*P* < 0.05), *n* = 15.

Meat color analysis showed that BPF affected selected L^*^, a^*^, and b^*^ values (Figure 2). In pectoral muscle, L^*^ was significantly lower in group II than in the control, groups I, and III (*P* < 0.05; Figure 2A). The a^*^ value was higher in groups I, II and III than in the control group (*P* < 0.05), with the highest value observed in group II (Figure 2B). Pectoral muscle b^*^ was not affected by treatment (*P* > 0.05; Figure 2C).

In thigh muscle, L^*^ did not differ among treatments (*P* > 0.05; Figure 2D). The thigh muscle a^*^ value was significantly higher in group II than in the control, I and III groups (*P* < 0.05), whereas the control and III were no significant difference (Figure 2E). The b^*^ value was significantly lower in groups II and III compared to the control group and group I, and group II was also significantly lower than group III (*P* < 0.05), while group I did not differ from the control group (Figure 2F). These results indicate that 200 mg/kg BPF improved redness and reduced yellowness in thigh muscle while modifying lightness and redness in pectoral muscle.

### 3.3 Effects of BPF on pectoral muscle fiber development

**Figure 3:**
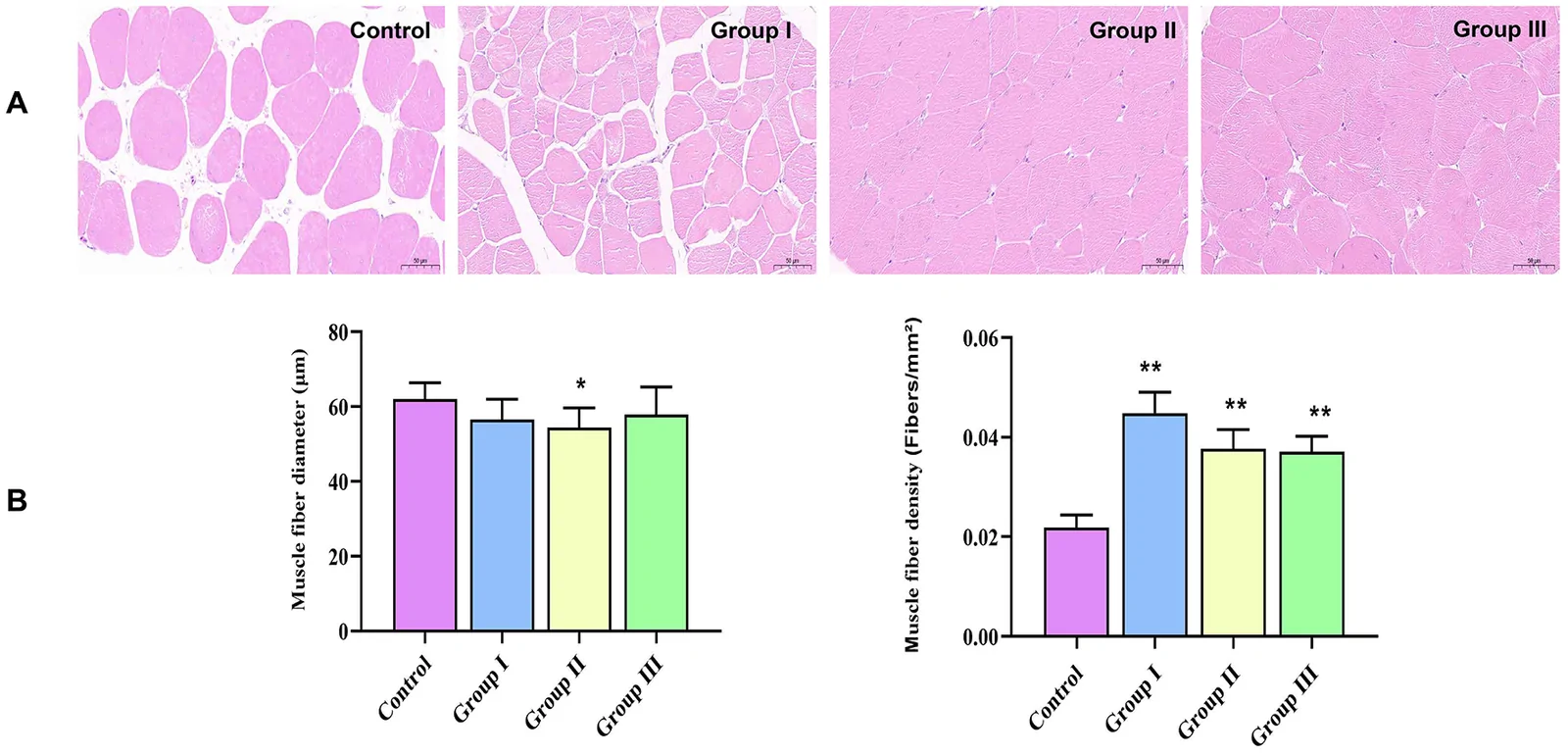
Effects of BPF on the growth and development of pectoral muscles in broilers. **(A)** Representative H&E-stained pectoral muscle sections from the control, groups I–III; scale bars = 50 μm. **(B)** Muscle fiber diameter and muscle fiber density of pectoral muscles. Six broilers were sampled for analysis of pectoral muscle development in each group. For each sample, three non-overlapping fields of 100,000 μm^2^ were analyzed. Error bars represent SEM of six independent samples. *P < 0.05 and **P < 0.01, *n* = 6.

Histological examination of pectoral muscle revealed that pectoral muscle fibers in the control group were larger and less densely arranged, whereas BPF-treated groups showed smaller fiber profiles and greater fiber density (Figure 3A). Quantitative analysis confirmed that pectoral muscle fiber diameter was significantly reduced in group II compared with the control group (*P* < 0.05), while groups I and III did not differ significantly from the control (Figure 3B). Muscle fiber density was significantly higher in all BPF-treated groups than in the control group (*P* < 0.01, Figure 3B).

### 3.4 Effects of BPF on thigh muscle fiber development

**Figure 4:**
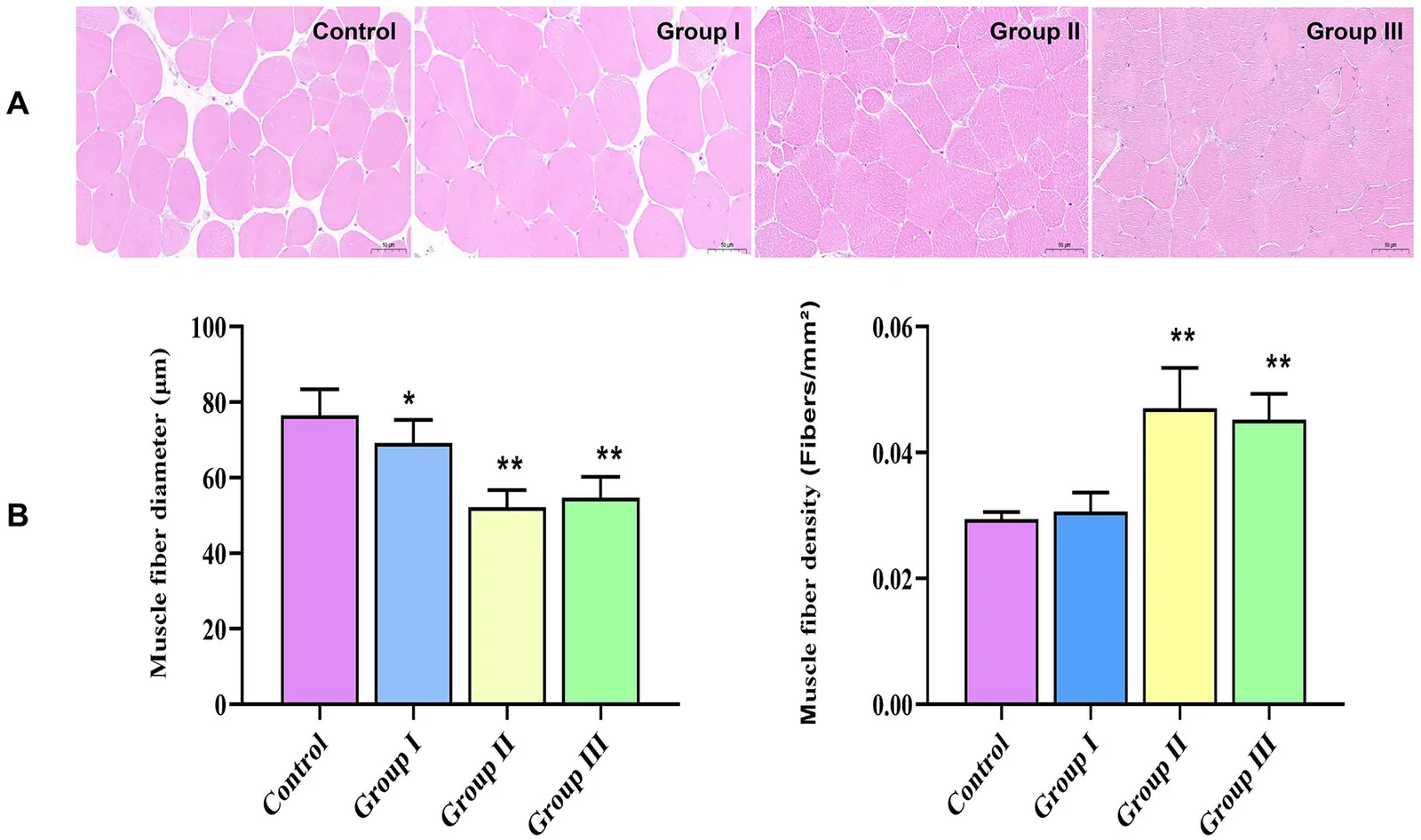
Effects of BPF on the growth and development of thigh muscles in broilers. **(A)** Representative H&E-stained thigh muscle sections from the control, groups I–III; scale bars = 50 μm. **(B)** Muscle fiber diameter and muscle fiber density of thigh muscles. Six broilers were sampled for analysis of thigh muscle development in each group. For each sample, three non-overlapping fields of 100,000 μm^2^ were analyzed. Error bars represent SEM of 6 independent samples. *P < 0.05 and **P < 0.01, *n* = 6.

Thigh muscle morphology also changed after BPF supplementation. Compared with the control group, BPF-treated groups showed smaller muscle fibers and a denser fiber arrangement (Figure 4A). Quantitative analysis showed that thigh muscle fiber diameter was significantly lower in groups I–III than in the control group (*P* < 0.05; Figure 4B). Muscle fiber density was significantly higher in groups II and III than in the control group (*P* < 0.01), whereas group I did not differ significantly from the control (Figure 4B).

### 3.5 Effects of BPF on antioxidant capacity in pectoral and thigh muscles

**Figure 5:**
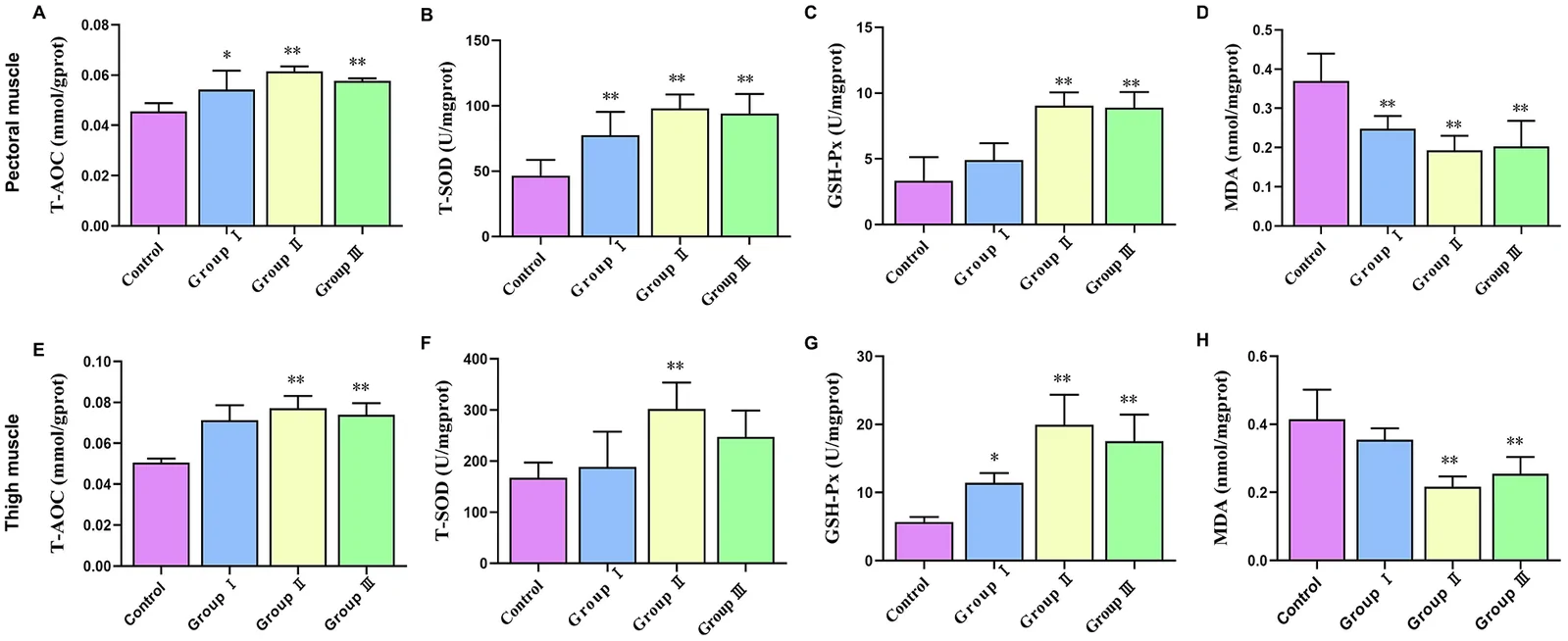
Effects of BPF on antioxidant capacity in broiler muscles. **(A)** T-AOC, T-SOD, GSH-Px, and MDA in pectoral muscle. **(B)** T-AOC, T-SOD, GSH-Px, and MDA in thigh muscle. Six broilers from each group were sampled for antioxidant capacity analysis. Error bars represent SEM of 6 independent samples. *P < 0.05 and **P < 0.01, *n* = 6. T-AOC: total antioxidant capacity; T-SOD: total superoxide dismutase; GSH-Px: glutathione peroxidase; MDA: malondialdehyde.

BPF supplementation enhanced antioxidant capacity in pectoral muscle (Figure 5A). Compared with the control group, T-AOC increased significantly in group I (*P* < 0.05) and highly significantly in groups II and III (*P* < 0.01). T-SOD activity was highly significantly increased in all BPF-treated groups (*P* < 0.01). GSH-Px activity was highly significantly increased in groups II and III (*P* < 0.01), whereas group I did not differ from the control. MDA content was highly significantly reduced in groups I–III compared with the control group (*P* < 0.01).

In thigh muscle, T-AOC was highly significantly increased in groups II and III (*P* < 0.01), whereas group I did not differ significantly from the control (Figure 5B). T-SOD activity was highly significantly increased only in group II (*P* < 0.01). GSH-Px activity increased significantly in group I (*P* < 0.05) and highly significantly in groups II and III (*P* < 0.01). MDA content was highly significantly reduced in groups II and III (*P* < 0.01), whereas group I showed no significant difference from the control. These findings indicate that 200 mg/kg BPF produced the most consistent antioxidant response in both muscles.

### 3.6 Non-targeted metabolomic responses in broiler muscles

**Figure 6:**
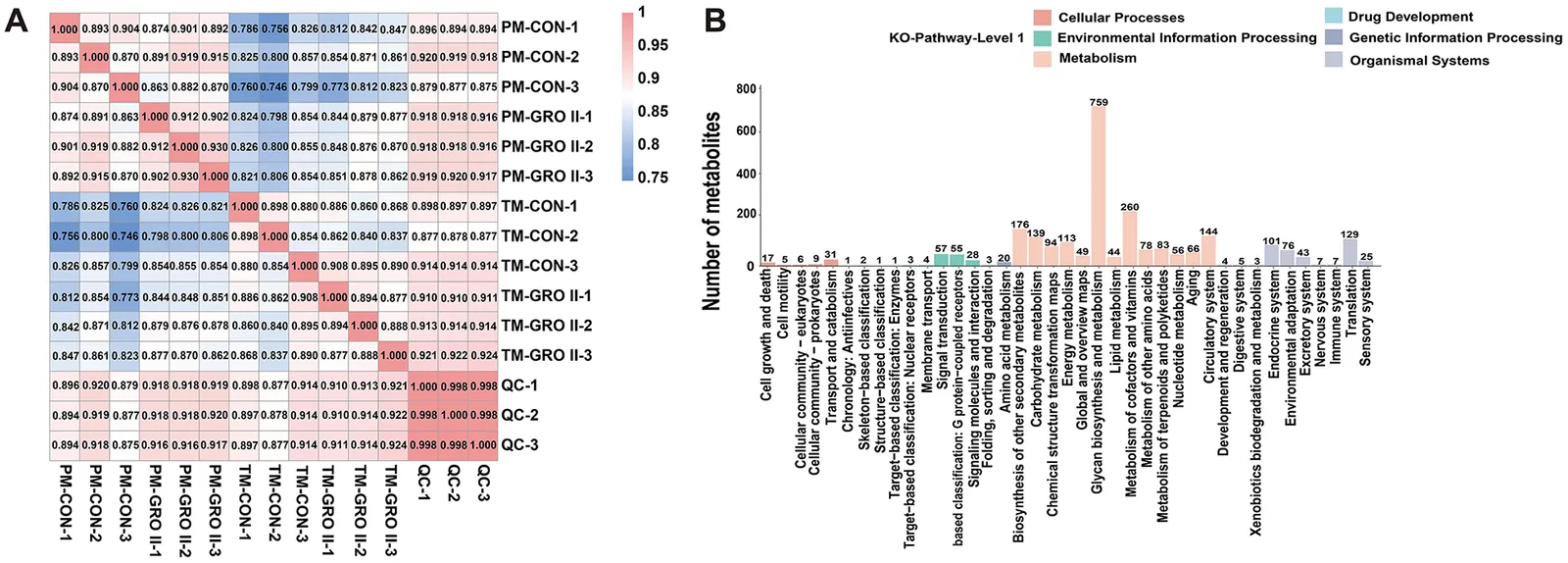
Effects of BPF on non-targeted metabolomics results of muscles in broilers. **(A)** Sample correlation heat map for metabolomics samples and quality-control samples from pectoral and thigh muscles. **(B)** KEGG Level 1 pathway annotation for pectoral and thigh muscle metabolites. PM-CON: pectoral muscle control group (*n* = 3); PM-GRO II: pectoral muscle group II (*n* = 3); TM-CON: thigh muscle control group (*n* = 3); TM-GRO II: thigh muscle group II (*n* = 3); QC: quality control (*n* = 3).

The LC-MS quality-control evaluation supported the reliability of the metabolomic dataset for pectoral and thigh muscles. In the sample correlation heat map, QC samples showed very high correlations, and biological replicates within each group showed broadly consistent metabolic profiles (Figure 6A). KEGG Level 1 annotation assigned the detected metabolites mainly to metabolism, organismal systems, environmental information processing, cellular processes, genetic information processing, and drug development categories (Figure 6B).

#### 3.6.1 Non-targeted metabolomic responses in pectoral muscle

**Figure 7:**
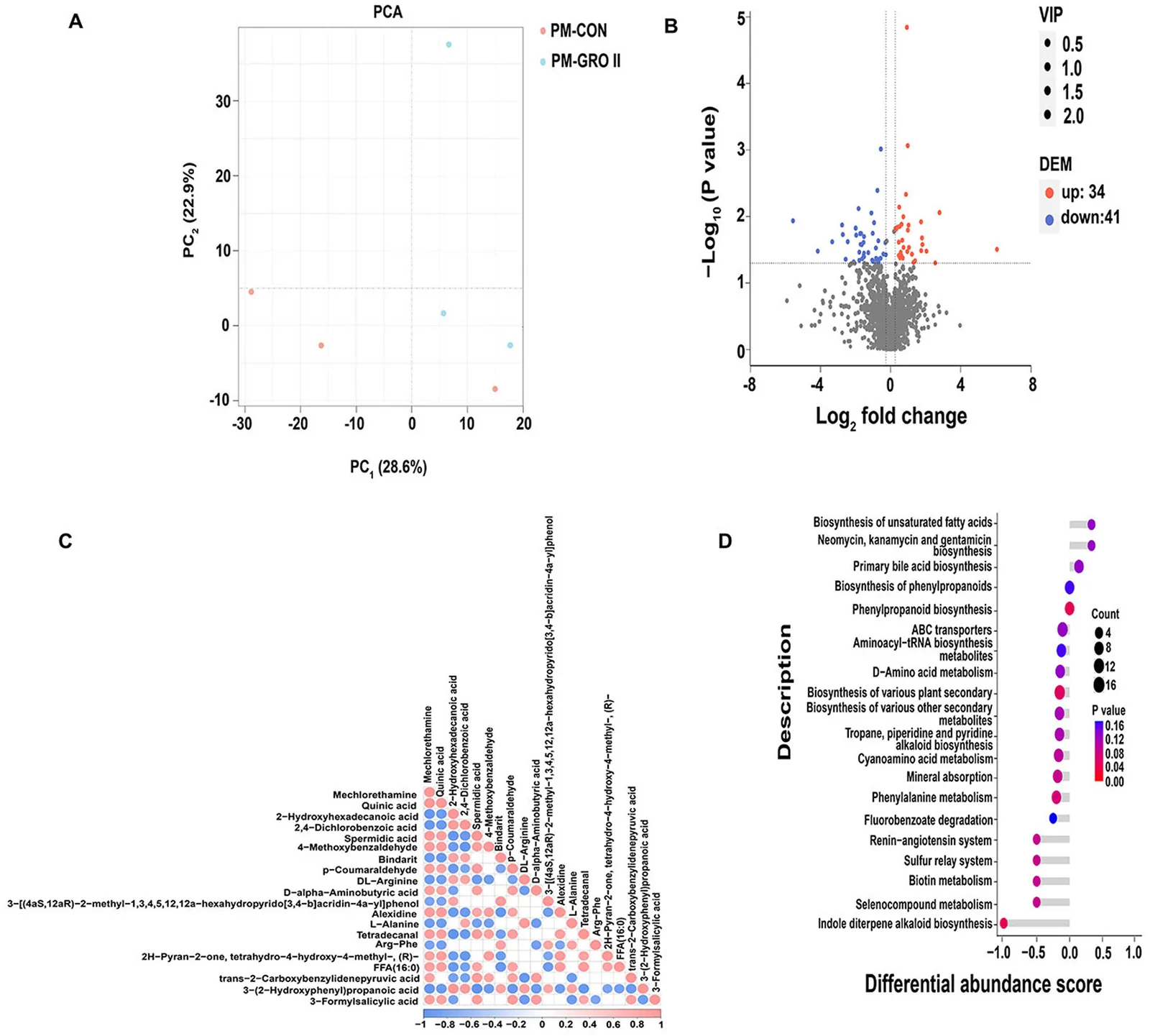
Effects of BPF on non-targeted metabolomics results of pectoral muscles in broilers. **(A)** PCA score plot of pectoral muscle samples. **(B)** Volcano plot of differential metabolites in pectoral muscles. **(C)** Correlation analysis of differential metabolites in pectoral muscles. **(D)** KEGG differential abundance score plot for pectoral muscles. PM-CON: pectoral muscle control group (*n* = 3); PM-GRO II: pectoral muscle group II (*n* = 3); QC: quality control (*n* = 3); VIP: variable importance in projection; DEM: differential metabolites.

In pectoral muscle, PCA showed a tendency toward separation between the pectoral control group and pectoral group II, indicating that 200 mg/kg BPF altered the pectoral muscle metabolomic profile (Figure 7A). Volcano plot analysis identified 75 differential metabolites in pectoral muscle, including 34 upregulated and 41 downregulated metabolites in pectoral group II compared with the pectoral control group (*P* < 0.05; Figure 7B).

Correlation analysis showed coordinated changes among several metabolites related to amino acid, organic acid, and lipid metabolism. Quinic acid, spermidic acid, p-coumaraldehyde, and FFA (16:0) were positively correlated with each other, whereas these metabolites were negatively correlated with DL-arginine, L-alanine, Arg-Phe, and 3-(2-hydroxyphenyl) propanoic acid (Figure 7C). Four pathways were significantly enriched: phenylalanine metabolism, D-amino acid metabolism, indole diterpene alkaloid biosynthesis, and phenylpropanoid biosynthesis (*P* < 0.05; Figure 7D).

#### 3.6.2 Non-targeted metabolomic responses in thigh muscle

**Figure 8:**
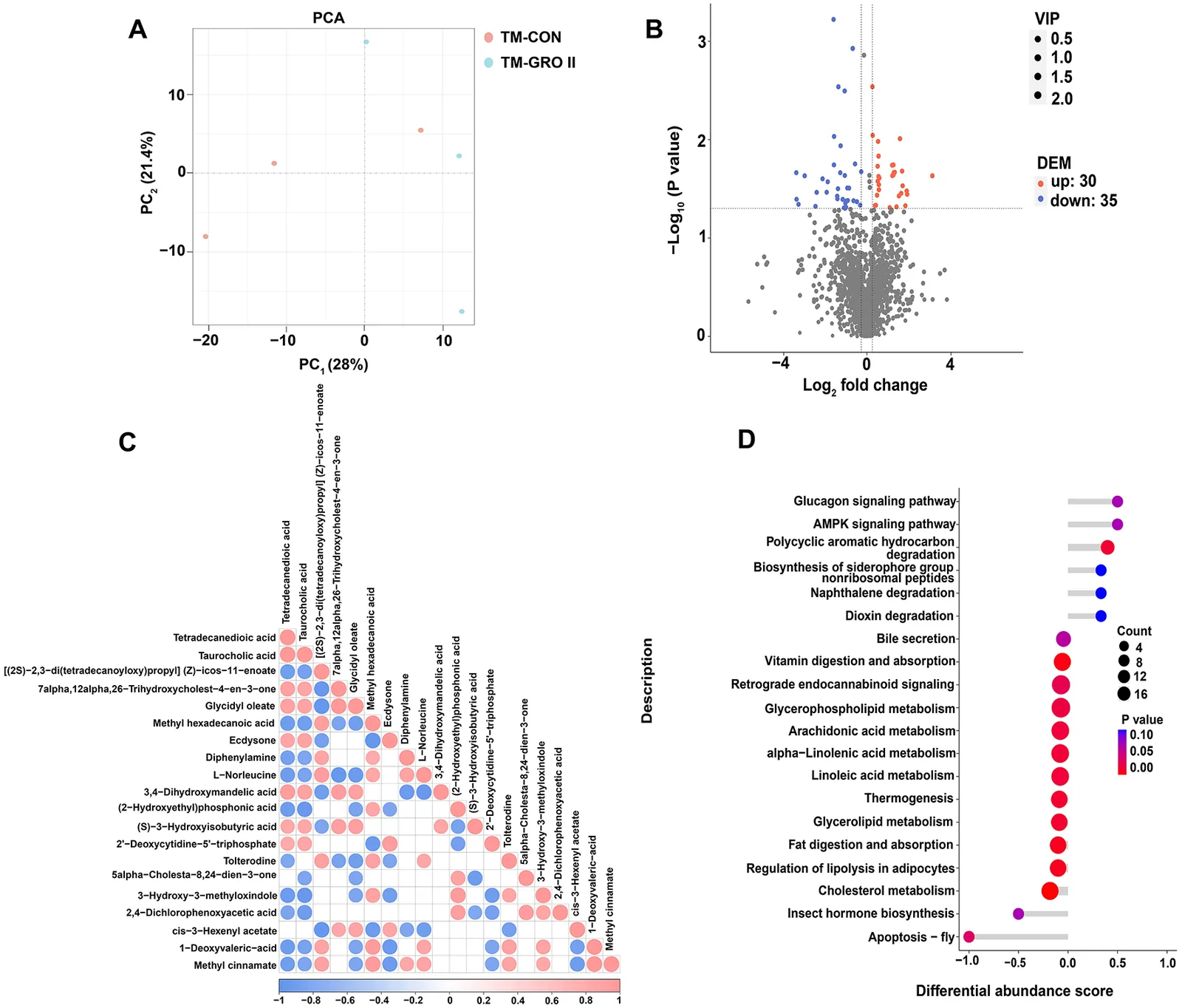
Effects of BPF on non-targeted metabolomics results of thigh muscles in broilers. **(A)** PCA score plot of thigh muscle samples. **(B)** Volcano plot of differential metabolites in thigh muscles. **(C)** Correlation analysis of differential metabolites in thigh muscles. **(D)** KEGG differential abundance score plot for thigh muscles. TM-CON: thigh muscle control group (*n* = 3); TM-GRO II: thigh muscle group II (*n* = 3); QC: quality control (*n* = 3); VIP: variable importance in projection; DEM: differential metabolites.

In thigh muscle, PCA showed a tendency toward separation between the thigh muscle control group and thigh muscle group II, indicating distinct metabolic profiles between the control and 200 mg/kg BPF groups (Figure 8A). Volcano plot analysis identified 65 differential metabolites in thigh muscle, including 30 upregulated and 35 downregulated metabolites in thigh muscle group II compared with the thigh muscle control group (*P* < 0.05; Figure 8B). The differential metabolites included compounds related to lipid and amino acid metabolism, including tetradecanedioic acid, L-norleucine, methyl hexadecanoic acid, and methyl cinnamate.

Correlation analysis showed that L-norleucine, methyl hexadecanoic acid, and methyl cinnamate were positively correlated with each other and negatively correlated with tetradecanedioic acid (Figure 8C). KEGG enrichment assigned thigh muscle differential metabolites to 52 pathways, including 12 significant pathways (*P* < 0.05; Figure 8D): cholesterol metabolism, regulation of lipolysis in adipocytes, fat digestion and absorption, glycerolipid metabolism, thermogenesis, linoleic acid metabolism, alpha-linolenic acid metabolism, arachidonic acid metabolism, glycerophospholipid metabolism, retrograde endocannabinoid signaling, vitamin digestion and absorption, and bile secretion.

## 4 Discussion

This study indicates that dietary BPF supplementation was associated with improvements in selected indices of broiler muscle quality, particularly at 200 mg/kg. BPF reduced selected water-loss indices, improved specific color traits, promoted a denser muscle fiber structure, enhanced antioxidant enzyme activities, reduced lipid peroxidation, and was accompanied by metabolomic differences related to amino acid and lipid metabolism. Together, these findings support the potential use of BPF as a phytogenic feed additive, while the metabolomic results should be considered exploratory rather than causal evidence.

Water-holding capacity, pH, shear force, and meat color are central indicators of meat quality. Muscle pH is linked to postmortem energy metabolism and protein function (14, 15), while drip loss and cooking loss reflect the ability of muscle proteins and fiber structures to retain water during storage and heating (16). Shear force is related to tenderness, collagen properties, and muscle fiber structure (17), and color parameters reflect the chemical state of myoglobin and hemoglobin (18). In the present study, 200 mg/kg BPF reduced pectoral cooking loss and thigh drip loss, altered fresh-muscle pH, increased a^*^ in selected muscles, and reduced thigh b^*^. These responses indicate improved water retention and color characteristics, but they do not support a conclusion that BPF improved tenderness because shear force was not significantly affected. Moderate postmortem acidification is part of normal meat aging, whereas excessive pH decline can impair protein function and water retention (19). Therefore, the observed pH decrease should be interpreted together with the water-loss and color results rather than as an independently beneficial effect.

The improvement in water-holding capacity may be related to changes in muscle fiber structure. Smaller fibers and higher fiber density can increase the uniformity of muscle architecture, although the present shear force data did not show a significant treatment effect. Previous research has shown that muscle fiber characteristics are closely related to poultry meat quality (11), and flavonoids such as quercetin and dandelion flavonoid extracts can improve meat quality by regulating antioxidant status and lipid oxidation (20, 21). In the present study, BPF reduced pectoral fiber diameter at 200 mg/kg and increased pectoral fiber density in all supplemented groups; it also reduced thigh fiber diameter in all supplemented groups and increased thigh fiber density at 200 and 300 mg/kg. These findings suggest that BPF supports muscle fiber refinement and fiber packing density during broiler growth, which may contribute to improved water retention without necessarily changing instrumental tenderness.

Oxidative stress is a major contributor to reduced meat quality in intensively reared broilers. Stress-induced reactive oxygen species can damage lipids, proteins, and cellular structures, whereas antioxidant enzymes such as SOD and GSH-Px help maintain redox homeostasis (10, 22–25). In this study, BPF increased T-AOC, T-SOD, and GSH-Px activities and reduced MDA content, especially at 200 mg/kg. Similar antioxidant-related improvements in broiler meat have been reported after quercetin-based dietary supplementation (26). The reduction in MDA indicates lower lipid peroxidation, which may help explain the improved water-holding capacity and color stability observed in BPF-treated muscles. These results are consistent with the antioxidant activity expected from flavonoid-rich feed additives.

The non-targeted metabolomic results provide exploratory association-level evidence for biological processes that may accompany the observed phenotypic changes in meat quality and antioxidant capacity. In pectoral muscle, BPF was associated with changes in metabolites related to amino acid metabolism, organic acid metabolism, and phenylpropanoid biosynthesis. Phenylalanine metabolism and D-amino acid metabolism may be related to protein turnover and flavor precursor availability, whereas phenylpropanoid-related metabolites may reflect antioxidant bioactivity. In thigh muscle, BPF was mainly associated with pathways involved in cholesterol metabolism, fatty acid digestion and absorption, glycerolipid metabolism, linoleic acid metabolism, alpha-linolenic acid metabolism, and arachidonic acid metabolism. Similar metabolomic remodeling has been reported in broiler muscle after supplementation with bamboo leaf flavonoids (27). Because pectoral and thigh muscles differ in fiber composition and metabolic demand, the muscle-specific metabolomic patterns may help explain why water-holding capacity, color, and antioxidant responses differed between the two tissues. However, these pathway associations require targeted metabolite quantification and functional validation before causal mechanisms can be established.

This study has several limitations. First, non-targeted metabolomics was conducted only on the control and 200 mg/kg BPF groups because this dose showed the most consistent meat quality and antioxidant responses, and only three biological replicates per group were used for each muscle type. Second, the pathway results are based on non-targeted, non-quantitative metabolomic annotation and enrichment analysis; targeted metabolite validation, enzyme activity assays, and transcriptomic or proteomic analyses are needed to confirm causal pathways and physiological relevance. Third, the study focused on a 42-day feeding period, and further work should evaluate whether BPF effects persist during meat storage and processing. Despite these limitations, the integrated phenotype and metabolome data indicate that 200 mg/kg BPF is a promising dietary level for improving selected broiler muscle quality traits.

## 5 Conclusion

Dietary BPF supplementation, particularly at 200 mg/kg, improved selected water-holding capacity indices, color traits, muscle fiber characteristics, antioxidant enzyme activities, and lipid peroxidation status in broiler muscles. Non-targeted metabolomic profiling further suggested associations with amino acid and lipid metabolic pathways, but these results should be interpreted as exploratory rather than definitive mechanistic evidence. These findings support further evaluation of BPF as a functional feed additive in broiler production.

## Data Availability

The data in the study are deposited in the NCBI Sequence Read Archive (SRA) under BioProject accession number PRJNA1475921.
